# Synthesis of nickel-sphere coated Ni-Mn layer for efficient electrochemical detection of urea

**DOI:** 10.1038/s41598-024-64707-z

**Published:** 2024-06-27

**Authors:** Nourhan Ezzat, Mahmoud A. Hefnawy, Sahar A. Fadlallah, Rabab M. El-Sherif, Shymaa S. Medany

**Affiliations:** 1 Bio Nanotechnology Department, Faculty of Nanotechnology, Cairo University, Giza, Egypt; 2 Chemistry Department, Faculty of Science, Cairo University, Giza, 12613 Egypt; 3 Biotechnology Department, Faculty of Science, Cairo University, Giza, 12613 Egypt

**Keywords:** Urea detection, Electrochemical sensor, Electrodeposition, Nickel manganese layer, Ni@NiMn/GC, Chemistry, Materials science

## Abstract

Using a trustworthy electrochemical sensor in the detection of urea in real blood samples received a great attention these days. A thin layer of nickel-coated nickel-manganese (Ni@NiMn) is electrodeposited on a glassy carbon electrode (GC) (Ni@NiMn/GC) surface and used to construct the electrochemical sensor for urea detection. Whereas, electrodeposition is considered as strong technique for the controllable synthesis of nanoparticles. Thus, X-ray diffraction (XRD), atomic force microscope (AFM), and scanning electron microscope (SEM) techniques were used to characterize the produced electrode. AFM and SEM pictures revealed additional details about the surface morphology, which revealed a homogenous and smooth coating. Furthermore, electrochemical research was carried out in alkaline medium utilizing various electrochemical methods, including cyclic voltammetry (CV), chronoamperometry (CA), and electrochemical impedance spectroscopy (EIS). The electrochemical investigations showed that the electrode had good performance, high stability and effective charge transfer capabilities. The structural, morphological, and electrochemical characteristics of Ni@NiMn/GC electrodes were well understood using the analytical and electrochemical techniques. The electrode showed a limit of detection (LOD) equal to 0.0187 µM and a linear range of detection of 1.0–10 mM of urea. Furthermore, real blood samples were used to examine the efficiency of the prepared sensor. Otherwise, the anti-interfering ability of the modified catalyst was examined toward various interfering species.

## Introduction

Urea is a byproduct of protein degradation and the metabolism of nitrogen-containing compounds. Exceeding a particular urea threshold in human body fluids (such as blood and urine) could harm the body^1–3^. It can cause nephritic syndrome, kidney or liver failure, abnormal urea levels in body fluids, and urinary tract blockage^4–6^. These infections can cause major health issues and even death if not diagnosed and treated promptly. Developing new and more accurate techniques for detecting urea in the body is vital. Improved urea detection technology can assist in early diagnosis and therapy, ultimately improving patient outcomes and quality of life^7–10^.

Urea sensors have various uses in fields such as medical diagnosis, food safety, and environmental monitoring^11,12^. Urea detection can be achieved using numerous detection methods, resulting in the development of many types of biosensors. These biosensors utilize optical^13^, thermal^14^, piezoelectric^15^, or magnetic measurements^16,17^.

Recently, many techniques, such as electrochemical sensors, have been used to detect urea. Electrochemical sensors have many advantages, such as affordability, detectability, and ease of experimentation^18–23^. They have proven useful in various sectors because they can detect and measure various analytes, including gases, ions, and biomolecules^24–29^.

Furthermore, the development of miniaturized and portable electrochemical sensors has transformed point-of-care diagnostics and personalized medicine. Additionally, these sensors have distinguished themselves from contemporary sensors on a large scale. When a target analyte is placed in an electrolyte, a redox reaction occurs, which results in changes in the electrical signal that are proportional to the concentration of the analyte. Based on the recorded signal, the electrochemical methods are classified into five types: aerometric, conductometric, impedimetric, potentiometric, and voltammetric^18^. In order to create new compositions using inorganic nanoparticles such as metal^30,31^, metal oxide^32–36^, metal hydroxide^37–39^, and metal sulfide^40–42^ have been employed recently for electrochemical detection applications. Electrochemical sensors made of Metal-Oxide (MO_x_) and binary metals are gaining attraction for successfully detecting electroactive biomolecules in various fields such as medical, environmental processes, energy-efficient systems, food safety, chemical, and agricultural industries^43–47^. The demand for such metal oxide-based biosensors is growing due to their ability to perform quick measurements and analysis while remaining versatile and trustworthy.

However, manganese dioxide (MnO_2_) has been specifically taken into consideration because it is less expensive, more abundant, and non-toxic than other inorganic oxides like cobalt, nickel, and vanadium^48–50^. One of the most significant functional metal oxides is manganese dioxide (MnO_2_), which has several uses in addition to its favorable electrochemical characteristics, low toxicity, inexpensive cost, and relative abundance. The controlled synthesis of MnO_2_ nano- and microcrystals with desired shapes has fueled fundamental studies in the past few decades thanks to advancements in nanoscience^51–54^. Their morphology significantly impacts the surface area, active site, and ion kinetics of materials, which can effectively influence their electrochemical performance^55^. Kulandaisamy et al., prepared MnO_2_ loaded RGO to enhance the detection of urea within linear range equaled to 5–100 μM and a limit of detection of 14.693 μM^56^. Furthermore, the combination between Ni and MnO_2_ reported by Zhu et al., that nickel doped to MnO_2_ nanosheet showed efficient actitivty to ward urea oxidation^57^.

In this work, we prepared a modified glassy carbon electrode with binary NiMn nanoparticles. Then, the surface was coated with NiO to enhance the activity of the electrode toward the electrochemical detection of urea in body fluids (especially the blood samples). The presence of binary catalysts like NiMn enhances the electronic properties of urea detection. else, the presence of an extra nickel layer promotes the activity of urea detection and nickel is considered as the most efficient surface of urea oxidation in an alkaline medium. Several electrochemical techniques have been used to characterize the prepared sensor like cyclic voltammetry, and electrochemical impedance spectroscopy (EIS). Else, anti-interference ability toward different species like ascorbic acid, glucose, dopamine, citric acid, and uric acid was investigated.

## Experimental

### Chemical and reagents

The following chemicals were utilized without additional purification: Nickel (II) sulfate (NiSO_4_.6H_2_O), Potassium permanganate (KMnO_4_), sulfuric acid (H_2_SO_4_) (98%), Urea, Sodium hydroxide (NaOH). All compounds used in this study are of analytical grade and were obtained from Sigma-Aldrich. The solutions were prepared using double distilled water.

### Instruments and devices

The structures of the **Ni@NiMn** samples were determined by analyzing them using X-ray diffraction (XRD) using Cu-Kα radiation (λ = 1.5406 Ǻ) on an Analytical X’Pert apparatus. The SEM (TESCAN VEGA 3, Czech Republic) was used to analyze the scanning electron microscope. The specimens were attached to aluminum stubs using adhesive carbon tape and then covered with a 150 s layer of gold (Au) using the Quorum Methods Ltd sputter coater (Q150t, England). The surface morphology was evaluated using high-resolution transmission electron microscopy (HR-TEM) with a JEOL JEM-2100 apparatus manufactured by Tokyo, Japan. The surface roughness and surface area of the produced **Ni@NiMn** were determined using atomic force microscopy using the Wet—SPM 9600 machine (Scanning Probe microscope, Shimadzu, Japan).

### NiMn and Ni precursors preparation

To prepare NiMn precursor, 0.395 g of KMnO_4_ (0.1 M) and 0.615 g of NiSO_4_ (0.1 M) were mixed in 15 mL of H_2_SO_4_ (0.1 M), then transferred to a 50.0 mL measuring flask and filled with distilled water to the mark. It was constantly stirred for a certain time to guarantee that NiMn was completely homogenates. For producing Ni precursor, dissolve 0.15 g of Na_2_SO_4_ (0.1 M) and 0.615 g of NiSO_4_ (0.1 M) in 50 mL of distilled water. Adding Na_2_SO_4_ to the Ni precursor increases the coating’s quality and surface adherence.

### Ni coated NiMn thin layer preparation (Ni@NiMn/GC)

NiMn layer was prepared by electrodeposition using cyclic voltammetry technique at 50 mV/s and potential window of (− 1.2 to 0.7 V) for 40 cycles. The electrodeposition procedure enabled controlled deposition of NiMn onto the glassy carbon electrode, resulting in a homogeneous and adherent coating. The cyclic voltammetry method was used to obtain the ideal thickness and composition of the NiMn layer. After NiMn layer preparation, the surface was activated in 1.0 M NaOH. Ni-coating was added to the dried NiMn layer using cyclic voltammetry for 30 cycles with a potential window that extended from (− 1.2 to 0.2 V). This process aimed to improve the surface qualities and guarantee that the obtained modified electrode had the required composition.

### Electrochemical measurements

The electrochemical measurement was carried out using a standard three-electrode setup, with Pt wire as the auxiliary electrode and Ag/AgCl/KCl (sat.) as the reference electrode. The modified Ni@NiMn/GC electrode was prompted as the working electrode. Phosphate Buffer Solution (PBS) used as an electrolyte at different pH values. Constant Potential Chronoamperometry (CA) and Electrochemical Impedance Spectroscopy (EIS) measurements were made using the Autolab PGSTAT128N potentiostat/galvanostat. Nova (Version 2.1, Metrohm Autolab, Utrecht, Netherlands) ran the fitted circuit. All potential measurements in this investigation were referred to Ag/AgCl/KCl (sat.) reference electrode. A constant AC voltage value was altered throughout the electrochemical impedance spectroscopy tests by employing an AC voltage amplitude of 10 mV and a frequency range of 1 × 10^4^ to 0.1 Hz.

## Result and discussion

### Surface and structure characterization

**Figure 1:**
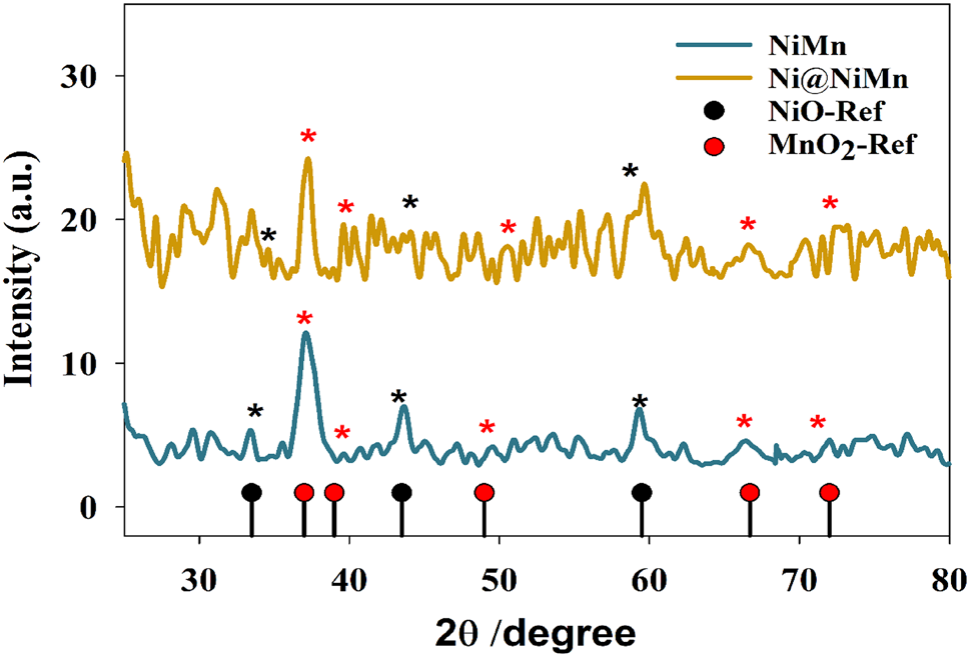
XRD of the NiMn and Ni@NiMn surfaces.

X-ray diffraction (XRD) technique was employed to determine the crystal structure and stability of the prepared NiMn and Ni@NiMn compounds. Figure 1 shows XRD of the modified materials NiMn and Ni@NiMn. However, the thin film of NiMn exhibits characteristic peaks for NiO, at 2θ equal 34, 43, and 59° for (111), (200) and (220) respectively (according to JCPDS card no. #47-1049)^58^. Additionally, the peaks attributed to MnO_2_ as observed at 2θ equal to 37^o^, 39^o^, 49^o^, 66^o^, 71^o^ corresponding to (211), (301), (411), (541), and (730) respectively (according to JSPDF 44-0141)^59^. Otherwise, the XRD was performed after an extra nickel layer was deposited. Whereas the peaks attributed to MnO_2_ have faded along with increase in peaks intensity at 2θ equal to 34, 43, and 59 that attributed to higher nickel concentration in sample.

**Figure 2:**
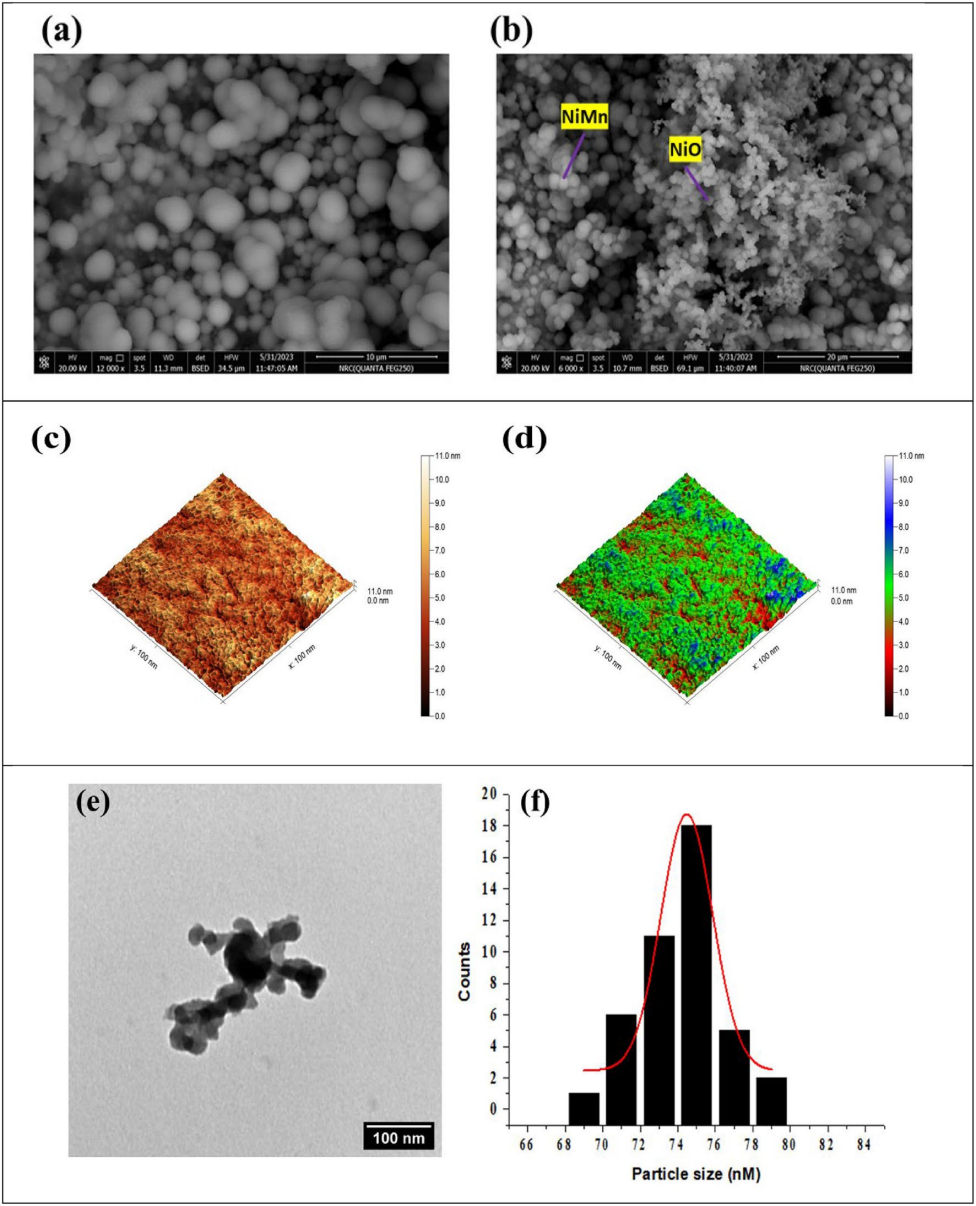

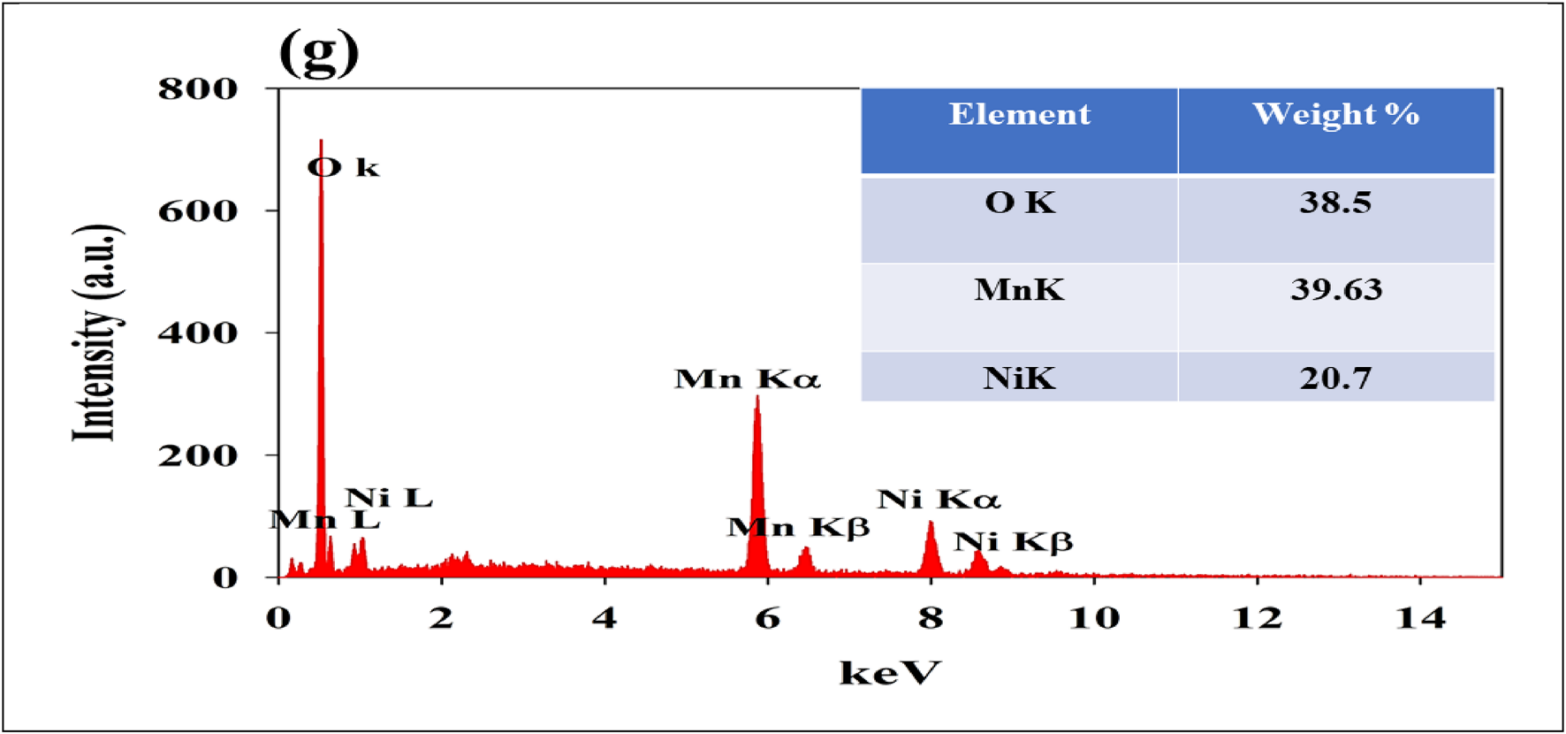
SEM of (**a**) NiMn; (**b**)Ni@NiMn surfaces; AFM (**c**,**d**) Ni@NiMn surface; (**e**) TEM of Ni@NiMn; (**f**) histogram of Ni@NiMn particle size distribution; and (**g**) Ni@NiMn EDX.

The surface morphology of the modified electrode was investigated using scanning electron microscopy (SEM). As represented in Fig. 2a-b, the surface of NiMn and Ni@NiMn was characterized as spheres distributed on electrode surface. Thus, the high cavities appeared on the surface facilitate the adsorption of the urea on electrode surface. Additionally, the presence of a nickel coating on the NiMn NPs further improves the conductivity and sensitivity of the network as depicted in Fig. 2c,d. Atomic force microscopy (AFM) was used to analyze the Ni@NiMn sample’s surface topography. As a result, predicted thick NiMn sheets covered with Ni are visible in 3D photos. AFM was used to evaluate the surface area and roughness as well. The surface area and mean roughness supplied were 0.82020 nm and 20050.8 nm^2^, respectively. These measurements indicate that the NiMn sheets coated with Ni have a relatively smooth surface with minimal roughness. This suggests that the coating process successfully achieved a uniform and well-adhered layer of Ni on the NiMn substrate. As represented in Fig. 2e, the crystal morphology of the Ni@NiMn using transmitted electron microscopy (TEM). However, the spherical shape was noticed with diameter ~ 70 nm. Furthermore, the distribution of particle size was estimated using TEM image and by using imageJ software. The gaussian fitting was used to find out the mean particle size. However, the provided mean particle size was ~ 75 nm (see Fig. 2f).

Figure 2g represents Ni@NiMn NPs composite components EDX. The EDX analysis confirms the presence of both nickel and manganese on the surface of the Ni@NiMn NPs composite. This suggests that the composite material is heterogeneous which possibly contributes to enhancing the electrochemical properties.

### Electrochemical sensing of urea

**Figure 3:**
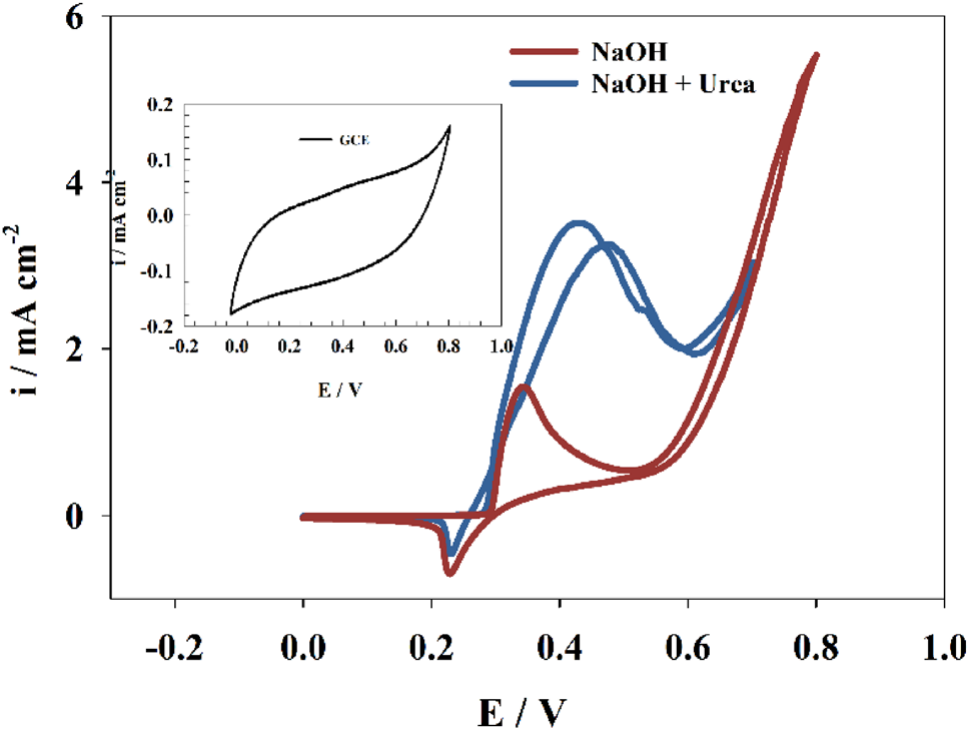
CV of the modified Ni@NiMn/GC in the presence and absence of 25 mM urea in 1.0 M NaOH at 20 mV/s. Inset figure is the CV of bare GCE in 1.0 M NaOH in the presence of 25 mM urea.

The cyclic voltammetry method was used to explore the electrochemical sensing of urea. Figure 3 represents the cyclic voltammograms of Ni@NiMn/GC in presence and absence of urea in 1.0 M NaOH as supporting electrolyte. Firstly, In the absence of urea, one nickel redox peak appears; the oxidation peak at 0.34 V caused by converting nickel hydroxide to nickel oxyhydroxide and the reduction peak at 0.22 V caused by converting nickel oxyhydroxide to nickel hydroxide, and the manganese peak may be overlapped with the high strength nickel peak current. Urea oxidation involves the conversion of urea into nitrogen gas (N_2_), carbon dioxide (CO_2_), and water (H_2_O). This complex process involves multiple electron transfer steps, and when urea is added, as in Fig. 3, two oxidation peaks are involved at (0.43, 0.48 V). One reduction peak of nickel overlapped with the manganese peak, which appeared at 0.23 V. In these CV curves, it is evident that urea concentration has a significant impact on the catalytic activity of Ni@NiMn/GC toward urea detection. The inset of Fig. 3 shows that the bare GCE has no activity toward urea oxidation in alkaline medium.

**Figure 4:**
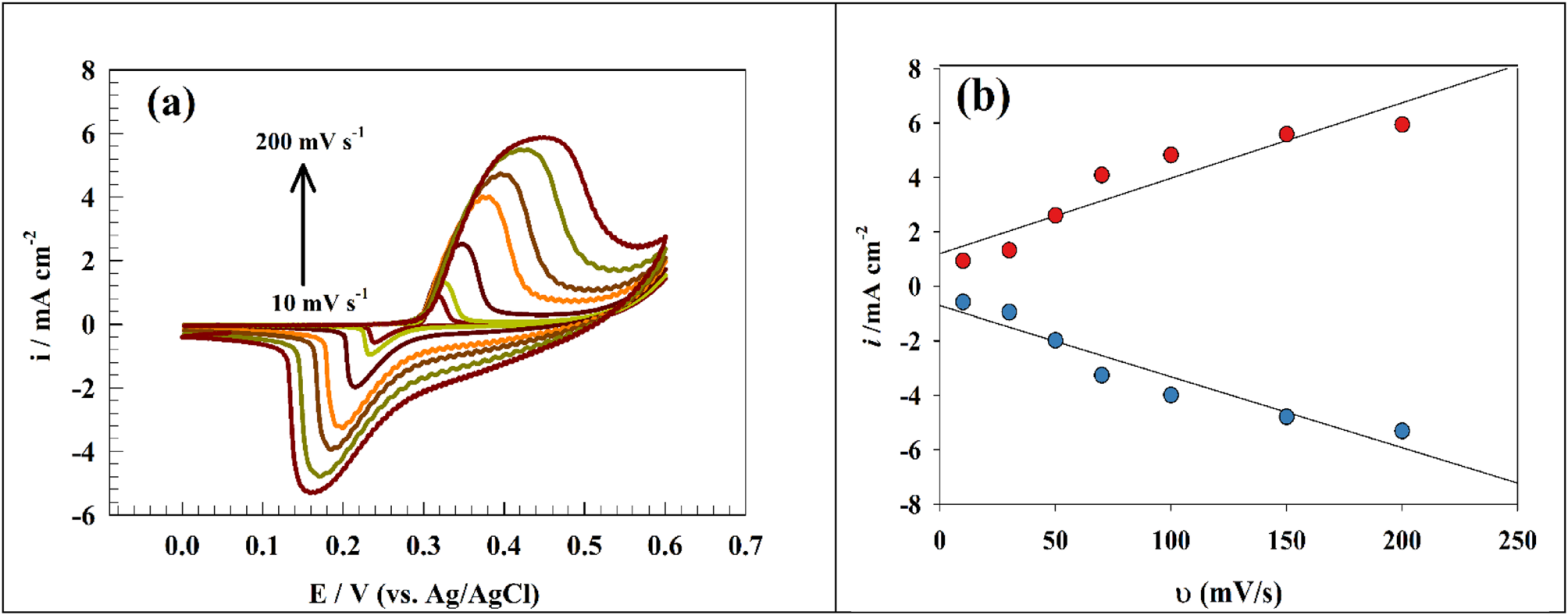
(**a**) CVs of Ni@NiMn/GC surface at a range of sweep rates. (**b**) The corresponding linear plot for redox current response versus the scan rate.

The electrode’s surface coverage describes how much a particular substance or material covers the electrode’s surface. In various electrochemical processes, it is a crucial component in influencing the effectiveness and performance of the electrode. Reaction rates, charge transfer kinetics, and general electrochemical behavior are the variables that the surface coverage might impact. More active sites can be found on the electrode due to greater surface coverage, which enhances the interaction between the material and the electrode. The enhanced contact produces a faster charge transfer rate, which raises current as a byproduct. It is vital to remember that there can be a threshold of diminishing returns at which further increases in surface coverage do not necessarily result in proportional increases in current. The surface coverage can be determined using the relationship that connecting the current with the scan rate (Eq. 1). Figure 4a depicts the cyclic voltammograms of Ni@NiMn/GC over sweep rate range (10–200 mV s^−1^) in a solution of one molar NaOH and 25 mM urea. It was noticed that oxidation peak potential shifted to more positive value as the scan rate increases which indicates that the process became less thermodynamically favored. The CVs show both oxidation and reduction processes concurrently when the scan rate is increased, indicating a reversible phenomenon. Figure 4b represents the dependence of the oxidation and reduction current peaks on the scan rate. The surface coverage can be calculated by the following equation^60^:

1 $$\Gamma = \, \left( {{\text{n}}^{{2}} {\text{F}}^{{2}} /{\text{4RT}}} \right) \, \left( {{\text{Ip}}/\upsilon {\text{A}}} \right)$$

where Γ stands for the surface coverage, n for the number of electrons (n = 6), F is Faraday’s number, R is the ideal gas constant, ʋ is the sweep rate**,** and T is the temperature in kelvin. The calculations estimated that the surface coverage is 7.207 × 10^−10^ mol/cm^2^ for Ni@NiMn/GC modified electrode.

**Figure 5:**
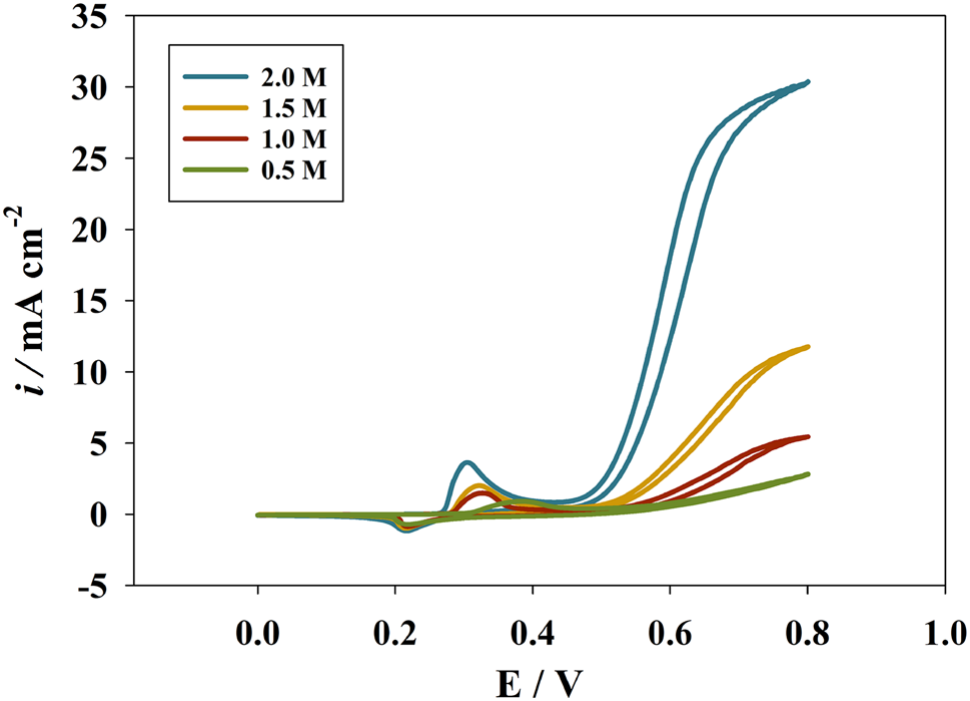
CVs of Ni@NiMn/GC in different NaOH concentrations at 20 mV/s.

The rate of nickel oxidation is affected by the electrolyte concentration because, as the concentration increases, the reaction rate accelerates, and the peak of the reaction moves to less positive. The presence of OH^-^ ions over the potential required for nickel ions activity can aid in oxidation. Figure 5 displays CVs of the modified Ni@NiMn/GC at different NaOH concentrations and 25 mM urea at a scan rate of 20 mV s^−1^. The shift in peak was observed for the oxidation peak, along with an increase in oxygen evolution rate. Furthermore, the move of oxidation peak towards more negative values implies a better environment for nickel oxidation at higher NaOH concentration according to the following equation^61–64^:

2 $$Ni(OH)_{2} + OH^{ - } \to NiOOH + H_{2} O + e^{ - }$$

The idea of Gibbs free energy may be used to illustrate this connection between thermodynamics and potential. As thermodynamics advances, the system becomes more stable, the potential energy drops, and ΔG rise. This rise in ΔG also denotes a fall in the reaction’s spontaneity. On the other hand, this improvement in kinetics is due to the increased rate of electron transfer. Additionally, higher current levels enhance the efficiency of reactant consumption, leading to faster reaction rates.

**Figure 6:**
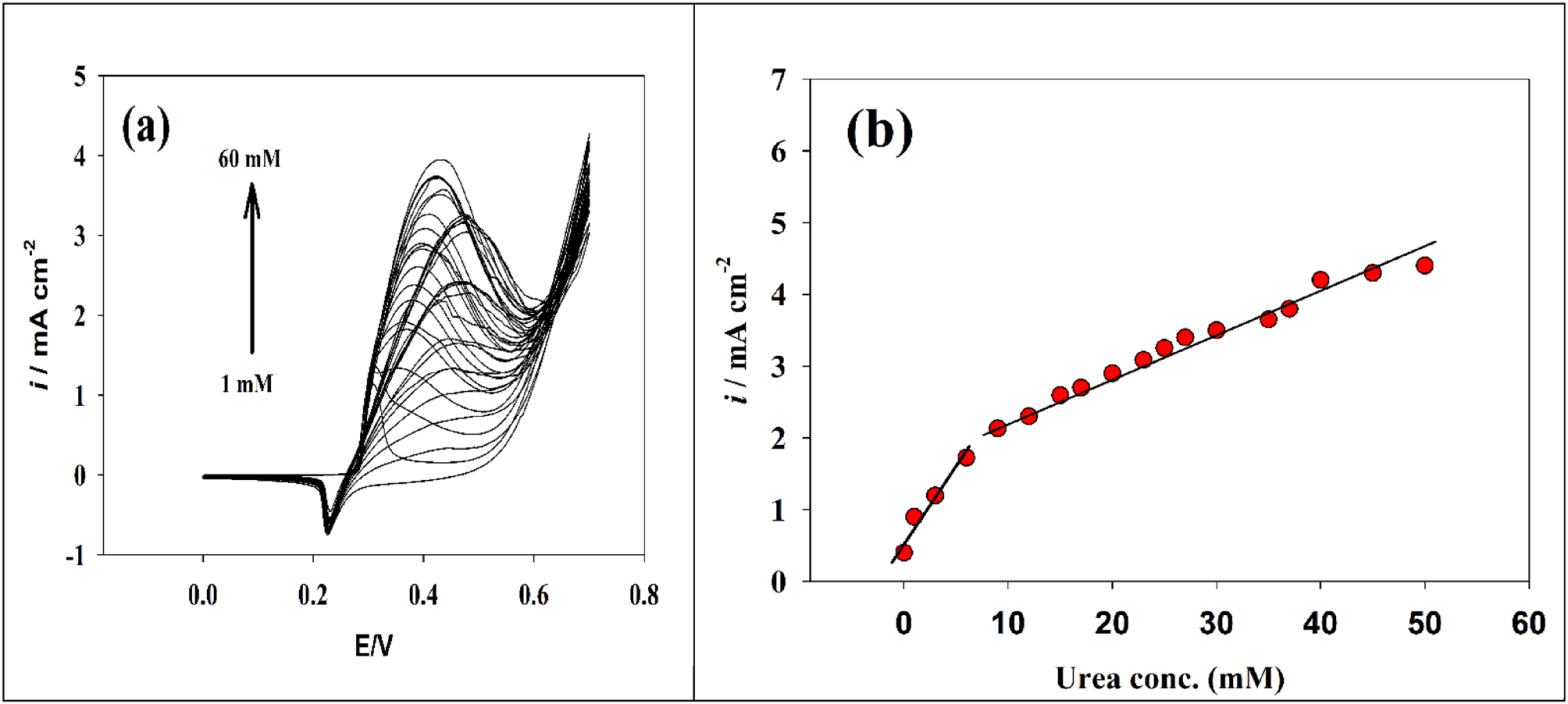
(**a**) Cyclic voltammetry of the modified Ni@NiMn/GC at various urea concentrations. (**b**) Linear plot of oxidation peak current versus different urea concentration.

Urea nitrogen levels in blood or serum typically from 5.0 to 20 mg/dl, or 1.8 to 7.1 mmol urea per liter. Due to typical fluctuations in protein intake, endogenous protein catabolism, hydration status, hepatic urea synthesis, and renal urea excretion, the range is broad. Excessive blood urea levels are referred to as uremia. Excessive urea levels in human physiological fluids (such as blood and urine) can damage the body. An accumulation of waste products in the blood that results from untreated kidney failure is known as uremia. Nausea, vomiting, weight loss, exhaustion, and trouble concentrating are some of the symptoms. Dialysis and kidney transplant surgery are two forms of treatment. Uremia is deadly if left untreated. Consequently, it was chosen to investigate the urea detection and sensitivity calibration curve over a concentration range. Therefore, the cyclic voltammetry (CV) method was utilized to investigate the effect of increasing the urea concentration (see Fig. 6a). In a solution of 1 M NaOH, the calibration curve and sensitivity of Ni@NiMn/GC electrode were tested across the concentration range of (1 to 60 × 10^−3^ M). The calibration curve for the modified electrode is depicted in Fig. 6b. The electrode demonstrated two linear urea detection ranges of 1–9 mM and 9–50 mM. The linear relationship was calculated using the following equations:

3 $${\text{Ip}}\;\left( {\mu {\text{A}}} \right)\; = \;0.180{\text{C}}_{{{\text{urea}}\left( {{\text{mM}}} \right)}} \; + \;0.585$$

4 $${\text{Ip }}(\mu A) \, = \, 0.051 \, C_{{{\text{urea}}\left( {{\text{mM}}} \right)}} + 1.8897$$

Moreover, the calibration curve’s slope was used to calculate the detection and quantization limits. The limit of detection is a method for calculating the least amount of analyte in a sample that can be detected. On the other hand, the limit of quantization is the lowest drug concentration that can be quantitatively detected using a specified accuracy and precision. The limit of detection (LOD) and limit of quantization (LOQ) for urea were derived using the following equations:

5 $${\text{LOD = 3}}\frac{{\text{s}}}{{\text{m}}}{,}\,{\text{and}}\, {\text{LOQ = 10}}\frac{{\text{s}}}{{\text{m}}}$$

where s is the standard deviation and m is the slope of the calibration curves.

LOD values for Ni@NiMn modified electrodes in a basic medium in low and high ranges of urea concentrations were 0.0187 and 0.054 mM, respectively. Furthermore, as compared to earlier research (see Table 1), the suggested sensor’s electrochemical characteristics are extremely stable, sensitive, and selective. LOQ for lower and higher concentration levels were 0.062 and 0.18 mM, respectively. The fact that the rate of the reaction rises with increasing urea concentration is one factor contributing to the increase in kinetic characteristics. The larger urea level makes more reactant molecules available, which raises the likelihood that current flow will increase.

**Table 1:** Comparison between the Ni@NiMn sensing performance and other reported catalysts.

Electrode	Linear range (mM)	Limit of detection (µM)	Method of detection	Detection technique	References
NiCo_2_O_4_ nanorods	0.1–10	6.0	Non-enzymatic	Cyclic voltammetry	^65^
(Ni-MOF) nanobelts	0.01–7.0	2.21	Non-enzymatic	Amperometric	^66^
Ni(OH)_2_/Mn_3_O_4_/rGO/PANi	0.03–3.3	16.3	Non-enzymatic	Cyclic voltammetry	^67^
Urease/ZrO_2_ thin film/Au	0.8–16.6	442	Enzymatic	Cyclic voltammetry	^68^
NiO–MoO_3_	0.2–1	0.86	Non-enzymatic	Cyclic voltammetry	^69^
Ni@NiMn	1–9	55.6	Non-enzymatic	Cyclic voltammetry	This work

### Electrochemical impedance spectroscopy

**Figure 7:**
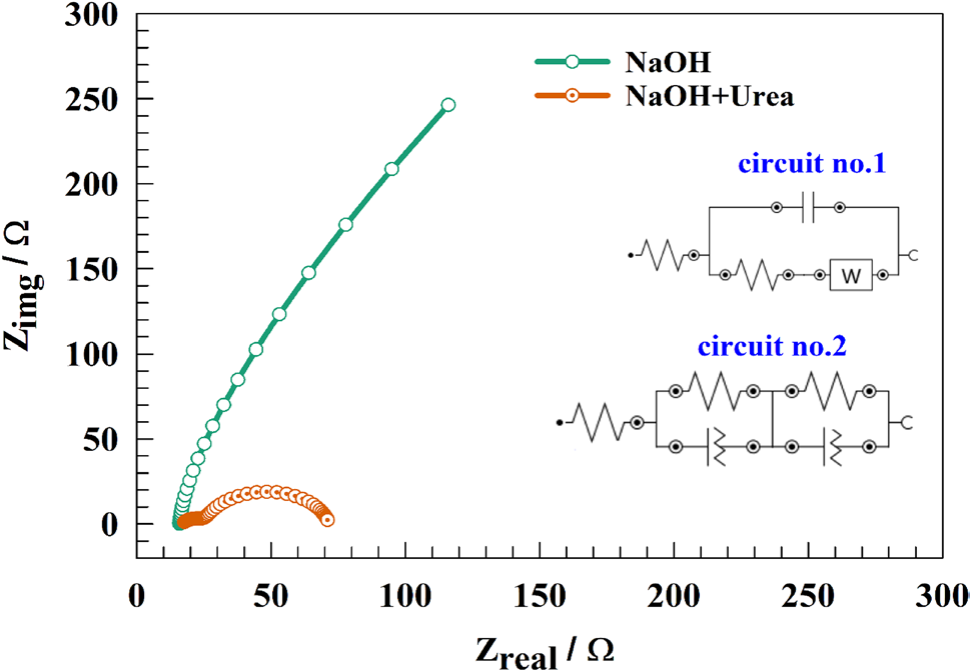
Nyquist plots of the modified Ni@NiMn/GC electrode and the fitting circuit of the modified electrode.

The charge transfer resistance was measured using electrochemical impedance spectroscopy technique. Figure 7 shows Nyquist plot of Ni@NiMn/GC electrode in a solution 1.0 M NaOH in absence and presence 25 mM urea at an AC constant potential of 0.55 V (vs. Ag/AgCl). Two semi-circles on the Nyquist plot show that the changed electrode is undergoing two separate charge transfer processes. The charge transfer process employing NiMn is represented by the inner circle, whereas the charge transfer process involving Ni coated is represented by the outside circle. Consequently, the results of electrochemical impedance spectroscopy (EIS) may be utilized to verify the conclusions reached by cyclic voltammetry (CVs). In line with the assertion are the analogous fitting circuits. The fitting electro equivalent circuit is depicted as an inset in Fig. 7 and Table 2 represents the fitting parameters. Whereas Ni@NiMn/GC was fitted using two different circuits namely circuit no.1 for NaOH and circuit no.2 for NaOH containing urea, respectively. Nyquist data may be modelled by the circuit of Rs, which stands for solution resistance; Rc, which stands for charge transfer resistance, C, which stands for the capacitance of the electrode layers, and the W (Warburg) element, which stands for the diffusion element. Higher urea detection efficiency can thus explain the lower urea impedance value in an alkaline medium. The EIS results provide further evidence to support the interpretations made from the CVs. Given the lower impedance value in an alkaline medium, the modified electrode could be more effective in detecting urea in this setting. This evidence supports the argument that higher resistance levels occur when urea is absent from the medium. Consequently, the absence of urea only one charge transfer resistance with Warburg element (diffusion element) was noticed. On the other hand, the EIS data in the presence of urea led to the formation of double charge transfer circuits which indicates that the oxidation of urea takes place on different oxidation layers inner layer (i.e., NiMn) and outer layer (i.e., Ni@NiMn).

**Table 2:** The fitting parameters for Ni@NiMn/GC electrode in the presence and absence of urea.

Electrolyte	Rs (Ω)	R_1_(Ω)	W	C_1_ (F)	R_2_(Ω)	C_2_(F)
NaOH	14.3	118	0.0024189	0.0000154	–	–
NaOH + Urea	16.7	24.8	–	0.0000376	71.6	0.0000477

### Real sample

The subsequent steps were employed during the actual sample analysis to quantify the quantity of urea: The concentrations of human blood serum samples were quantified and after that stored at a temperature of 4 °C in sterile vials within a clinical laboratory setting. To mitigate matrix complexity before analysis, the materials underwent a 200-fold reduction using NaOH with a concentration of 1.0 M. To prepare a stock solution with a concentration of 50 mmol/L, urea was dissolved in blood serum without undergoing any pre-treatment procedures. The initial point for standard additions was utilizing a stock solution within a 10 mL volume of 1.0 mol L^−1^ NaOH. Subsequent observations were conducted under ambient temperature conditions, specifically at 20 °C. The experiment focused on determining the capacity of a modified electrode to detect urea at various concentrations, including low and high levels. Following the production of the real sample, the urea electrochemical sensing method was used to assess the urea detection and sensitivity calibration curve in the concentration range.

**Figure 8:**
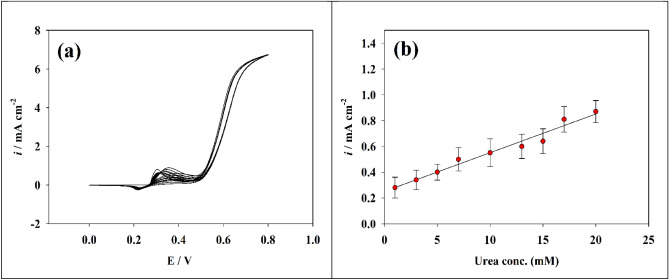
(**a**) CVs of the modified Ni@NiMn/GC in blood. (**b**) The calibration curve for various urea concentrations in blood.

To study the effect of increasing the urea concentration, the cyclic voltammetry (CV) technique was used in blood sample (Fig. 8a). In Fig. 8b, the calibration curve for the modified electrode is shown. The electrode has a linear urea detection range of 1.0–21 mM. The following equation was used to determine the linear relationship:

6 $${\text{Ip }}\left( {{\mu A}} \right){ = 0}{\text{.03 C}}_{{\text{urea(mM)}}} { + 0}{\text{.25}}$$

Furthermore, the detection and quantization limitations were calculated using the calibration curve’s slope. In blood, the LOD value for Ni@NiMn/GC modified electrodes was 0.12 mM. Lower and higher concentration levels had LOQs of 0.4 mM.

Using blood samples generated for the non-enzymatic urea sensor’s analysis, the usefulness of this sensor was demonstrated. Table 3 represents the reported results from the three times of real sample analysis. The suggested non-enzymatic urea sensor has been demonstrated to be reliable and practical based on its capacity to accurately determine urea from blood. The encouraging urea determination from actual blood sample is confirmed by the low relative standard deviation (RSD) values of less than 1.0%. The RSD (%) was calculated by dividing the standard deviation on the average of the quantified urea data from three consecutive cycle urea sensing studies, multiplied by 100%.

**Table 3:** Recovery of urea detection in blood sample.

Sample	Added (mM)	Found (mM)	Recovery
blood	–	3.4	–
	1.0	3.3	97%
	1.5	5	102%
	2.0	5.3	98.2%
	2.5	5.9	100%

### Interference

**Figure 9:**
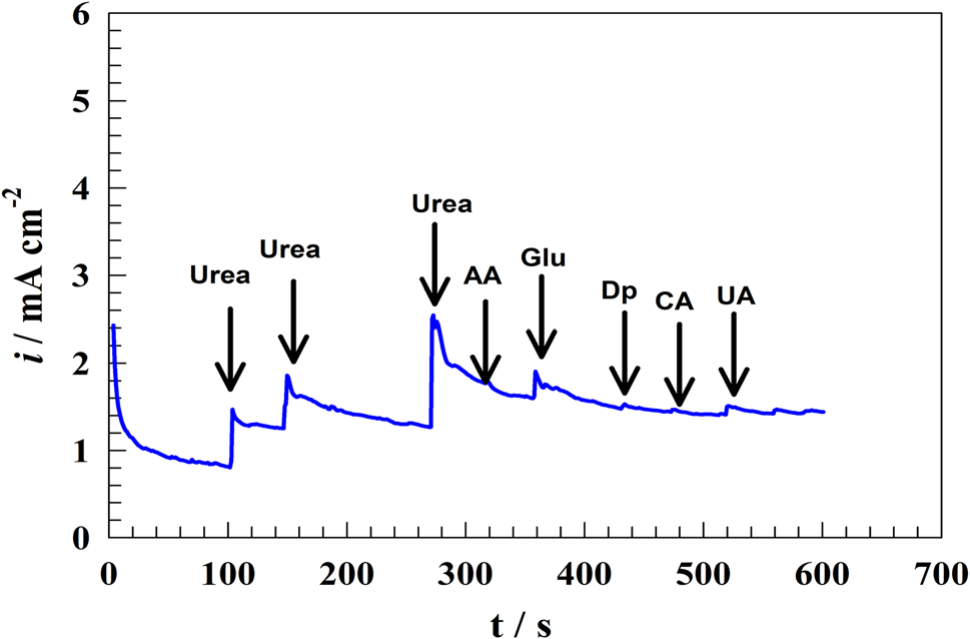
Chronoamperogram of modified Ni@NiMn/GC electrode in the presence of different interfering species.

It is essential to assess the suggested sensor’s capacity to distinguish between the investigated analyte and the interfering species in human fluids selectively and simultaneously. Human bodily fluids include common interfering species: ascorbic acid (AA), glucose (Glu), dopamine (Dp), Citric acid (CA), and uric acid (UA). It may be not easy to accurately detect other substances under study when interfering species are present. Therefore, when creating a suggested method for detecting urea in biological samples, it’s crucial to consider and address their potential influence. In order to test the modified electrode selectivity in the presence of urea, competing agents were added one after the other during the urea detection process. Figure 9 shows the chronoamperogram of the modified Ni@NiMn/GC in 1.0 M NaOH at a constant potential of 0.6 V. The urea was added to ensure the activity of the electrode surface. Then, different interfering species were added to the surface. A slight increase in the detected current was observed for glucose and uric acid. Whereas the oxidation potential of glucose and uric acid are near to the urea oxidation potential. Since the electrocatalytic material’s nature mostly determines the selectivity of non-enzymatic sensors, materials’ electrocatalytic properties have already been evaluated and investigated for a narrow range of applications, as previously stated. Because of their great selectivity for a specific application, this has led to much research into the production of highly electrocatalytic materials.

## Conclusion

A simple and accurate electrochemical sensor based on coating the GC electrode surface by two sequential layers of NiMn and Ni nanoparticles is proposed to investigate urea in blood samples with LOD equaled to 0.12 mM. LOD values for Ni@NiMn modified electrodes in a basic medium in low and high ranges of urea concentrations were 0.0187 and 0.054 mM, respectively. The exceptional synergism of NiMn NPs and Ni-coated nanoparticles and their special features as modifiers enhance the urea electrochemical current response when present in blood samples with interfering ions and compounds. Additionally, the Ni@NiMn/GC electrode can accurately detect urea in actual samples. The presence of various compounds in real blood samples characterizes the ability of electrodes to prevent interference.

## Data Availability

The datasets used and/or analysed during the current study are available from the corresponding author on reasonable request.
